# Global trends in COVID-19

**DOI:** 10.1016/j.imj.2021.08.001

**Published:** 2022-03-04

**Authors:** Chuan-Min Zhou, Xiang-Rong Qin, Li-Na Yan, Yuan Jiang, Xue-Jie Yu

**Affiliations:** a Department of Infectious Diseases, Zhongnan Hospital, Wuhan University, Wuhan, 430071, Hubei Province, China; b State Key Laboratory of Virology, School of Health Sciences, Wuhan University, Wuhan, 430071, Hubei Province, China; c Department of Clinical Laboratory, the Second Hospital of Shandong University, Jinan, 250033, Shandong Province, China

**Keywords:** COVID-19, SARS-CoV-2, Herd immunity, Case fatality rate, Breakthrough infection, Delta variant, Omicron variant, Vaccine

## Abstract

The pandemic COVID-19 is certainly one of the most severe infectious diseases in human history. In the last 2 years, the COVID-19 pandemic has caused over 418.6 million confirmed cases and 5.8 million deaths worldwide. Young people make up the majority of all infected COVID-19 cases, but the mortality rate is relatively lower compared to older age groups. Currently, about 55.04% individuals have been fully vaccinated rapidly approaching to herd immunity globally. The challenge is that new SARS-CoV-2 variants with potential to evade immunity from natural infection or vaccine continue to emerge. Breakthrough infections have occurred in both SARS-CoV-2 naturally infected and vaccinated individuals, but breakthrough infections tended to exhibit mild or asymptomatic symptoms and lower mortality rates. Therefore, immunity from natural infection or vaccination can reduce SARS-CoV-2 pathogenicity, but neither can completely prevent SARS-CoV-2 infection/reinfection. Fortunately, the morbidity and mortality of COVID-19 continue to decline. The 7-day average cumulative case fatality of COVID-19 has decreased from 12.3% on the February 25, 2020, to 0.27% on January 09, 2022, which could be related to a decreased SARS-CoV-2 variant virulence, vaccine immunization, and/or better treatment of patients. In conclusion, elimination of SARS-CoV-2 in the world could be impossible or at least an arduous task with a long way to go. The best strategy to prevent COVID-19 pandemic is to expand inoculation rate of effective vaccines. As the population reaches herd immunity, the mortality rate of COVID-19 may continue to decrease, and COVID-19 could eventually become another common cold.

## Highlights

• COVID-19 vaccines has been vaccinated globally for reaching herd immunity.
• Emergence of SARS-CoV-2 Omicron variants enhance the risk of breakthrough infections.
• SARS-CoV-2 Omicron variants tend to exhibit lower virulence and pathogenicity.
• The case fatality of COVID-19 keeps decreasing with decreased SARS-CoV-2 virulence, improved medical treatments, and distribution of COVID-19 vaccines.

## 1 Introduction

Coronaviruses are a group of enveloped RNA viruses with unsegmented, positive-stranded RNA genomes that are classified in the order *Nidovirales*, the family *Coronaviridae*, and the subfamily *Coronavirinae*. Based on serological evidence and genomic structures, the subfamily *Coronavirinae* is subdivided into the 4 genera *Alphacoronavirus, Betacoronavirus, Gammacoronavirus*, and *Deltacoronavirus.* Actually, coronaviruses have been discovered all over the world, accompanying with that most of us will be infected by a coronavirus at some point at least once in our lives. In general, coronaviruses cause mild to moderate respiratory and intestinal infections in vertebrates [1]. To date, 7 human coronaviruses have been detected with 4 human coronaviruses causing common cold including OC43 (β-CoV), HKU1 (β-CoV), 229E (α-CoV), and NL63 (α-CoV), and 3 human coronaviruses causing severe respiratory diseases including severe acute respiratory syndrome coronavirus (SARS-CoV) (β-CoV) [2,3], Middle East respiratory syndrome coronavirus (MERS-CoV) (β-CoV) [4], and SARS-CoV-2 (β-CoV) [5].

After the first report of COVID-19 cases in late 2019, SARS-CoV-2 spread rapidly around the world. The World Health Organization (WHO) declared COVID-19 as a serious public health emergency of international concern on January 30, 2020, and a pandemic situation on March 11, 2020. To date, the COVID-19 pandemic has wreaked havoc around the world for 2 years. Globally, over 418.6 million confirmed COVID-19 cases and 5.8 million deaths have been reported, and that is certainly an undercount because of many lower income areas still lack enough medical resources. According to the Global Economic Prospects report, economic growth would slow down under COVID-19 pandemic and was expected to decline markedly from 5.5% in 2021 to 4.1% in 2022, and 3.2% in 2023. We are still trapped in the COVID-19 pandemic. Fortunately, the good news is that, despite the constant appearance of new SARS-CoV-2 variants, the case fatality rate (CFR) of COVID-19 keeps decreasing, which could be associated with the immunization with COVID-19 vaccine globally, the improved medical treatment for COVID-19, and the decrease virulence of SARS-CoV-2 variants, importantly, that do not mean we quit battling against COVID-19. Proper policies to control and prevention COVID-19 pandemic precisely are still important until the moment comes. However, there is still so much we do not know about SARS-CoV-2, and we still have a long way to go with SARS-CoV-2. More efforts are urgently needed to understand SARS-CoV-2 and control the outbreak of COVID-19. We must face the reality that SARS-CoV-2 could continue to coexist with humans on Earth for a long time.

## 2 The incidence of COVID-19

**Fig. 1:**
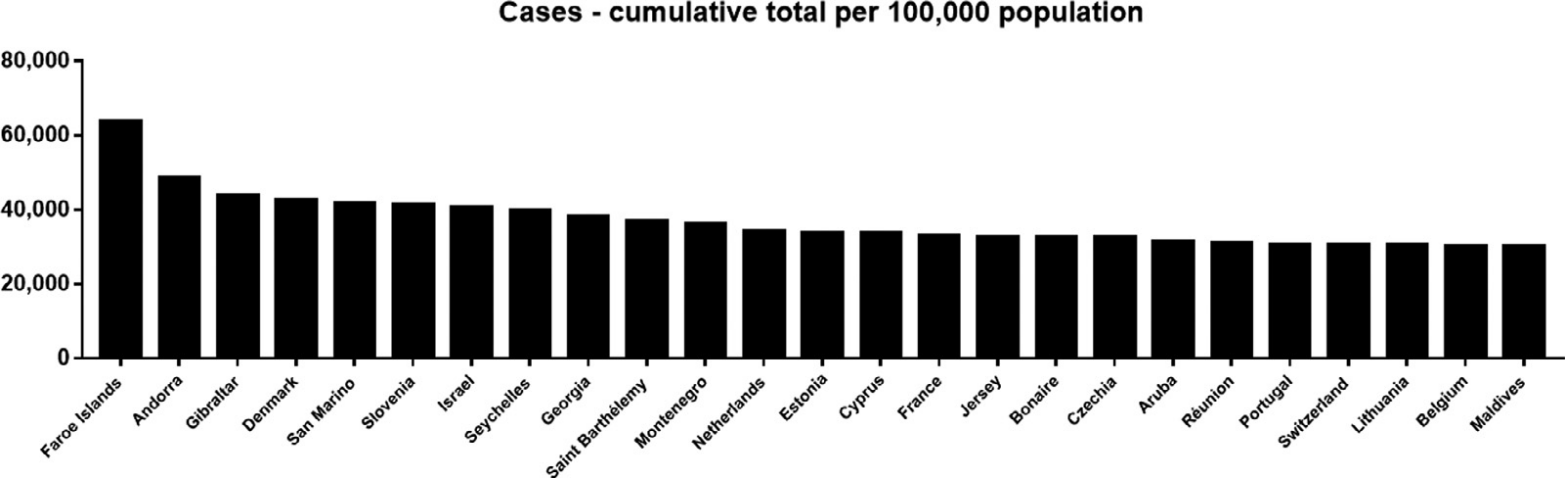
Top 25 Countries with cumulative cases per 100,000 population as of February 18, 2022 (https://covid19.who.int/). (Color version of figure is available online).

**Fig. 2:**
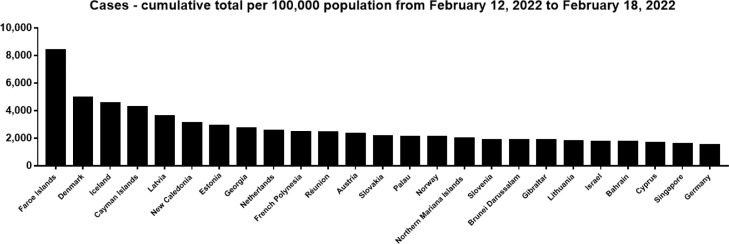
Top 25 Countries with cumulative cases per 100,000 population from February 12, 2022 to February 18, 2022 (https://covid19.who.int/). (Color version of figure is available online).

Reported COVID-19 cases may be just the tip of the iceberg, as more than 80% of people with COVID-19 are asymptomatic or mild, which may go unrecognized and unreported [6]. As of February 18, 2022, countries with more than 10 million reported cases include the United States (77,521,589), India (42,780,235), Brazil (27,806,786), France (21,436,445), The United Kingdom (18,499,062), Russian Federation (15,020,573), Turkey (13,265,374), Germany (13,255,989), Italy (12,323,398), and Spain (10,778,607). Countries with better and applicable diagnostic facilities, higher population size, and/or older population may report more cases. Hence, the incidence rate of COVID-19 may better reflect the sensitivity of the population to SARS-CoV-2 and the level of diagnosis. Based on the current information, countries with a high incidence rate of COVID-19 per 100,000 population have been reported mainly in Europe, such as Faroe Islands (63,628.36/100,000), Andorra (48,562.74/100,000), Gibraltar (43,904.90/100,000), Denmark (42,649.84/100,000), San Marino (41,684.84/100,000), Slovenia (41,421.74/100,000), and Israel (40,620.43/100,000) (Fig. 1). In addition, countries with a high incidence rate of COVID-19 per 100,000 population in last 7 days have also been reported mainly in Europe, such as Faroe Islands (8,374.09/100,000), Denmark (4,926.24/100,000), Iceland (4,541.46/100,000), Latvia (3,594.01/100,000), Estonia (2,893.66/100,000), Georgia (2,711.79/100,000), and Netherlands (2,526.65/100,000) (Fig. 2). The high incidence in Europe may be due to the aging of the European population and the high level of diagnosis.

## 3 The case fatality rate of COVID-19

People around the world did not suffer from SARS-CoV-2 before 2019 and are therefore highly susceptible to SARS-CoV-2. According to the WHO reports, the cumulative CFR in the early phase of COVID-19 was 5.6% in mainland China and 15.2% outside China [7]. Such a high mortality rate in the early phase of the outbreak of COVID-19 might be due to the high virulence of SARS-CoV-2 and the lack of knowledge of proper patient management. However, the initial high CFR of COVID-19 could also be the iceberg phenomenon of infectious diseases where only patients with severe disease course were detected and a large percentage of mild and subclinical cases who did not want to go to hospital remained hidden and were not counted as denominators in the severe epidemic areas in the initial phase of the COVID-19 outbreak.

**Fig. 3:**
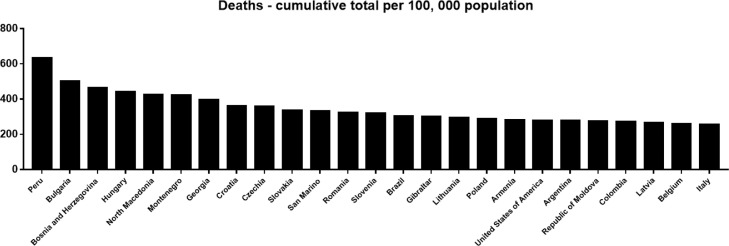
Top 25 Countries with cumulative deaths per 100,000 population as of February 18, 2022 (https://covid19.who.int/). (Color version of figure is available online).

The reported CFR of COVID-19 varies widely among different countries and regions in the world. The countries with the higher CFR (≥5) of COVID-19 (cumulative total deaths/ cumulative total cases) concentrated in Eastern Mediterranean and Americas, including Yemen (18.0%), Sudan (6.4%), Peru (6.0%), Mexico (5.9%), Syrian Arab Republic (5.7%), Somalia (5.1%), and Egypt (5.1%), which to some content reveals the inadequacy of the world's interconnected medical systems in coping with this unknown severe coronavirus challenge. In addition, cumulative deaths based on a population of 100, 000 show that many European and American countries exhibit high mortality rates. Of note, country with the highest case fatality of COVID-19 is Peru (633.23/100,000) (Fig. 3). The high mortality in European countries may be due to the aged population. The countries with a reported high case fatality of COVID-19 such as Yemen, Sudan, Mexico, Syrian Arab Republic, Somalia, and Egypt do not have a high mortality rate for COVID-19, suggesting that high CFR in these countries may be due to ignorance or misdiagnosis in mild patients or improper treatment of patients. Judging from the high infection rate, the number of reported cases in the area with a high incidence of COVID-19 may be far lower than the actual number of infections [8]. The CFR reported by some countries may be inaccurate due to neglect of patients with mild disease [20]. The exact denominator of COVID-19 cases remains unclear. Our research group analyzed the seroprevalence of SARS-CoV-2 in Wuhan City and found that approximately 1.68% of individuals in Wuhan City were seropositive to SARS-CoV-2 [9].

**Fig. 4:**
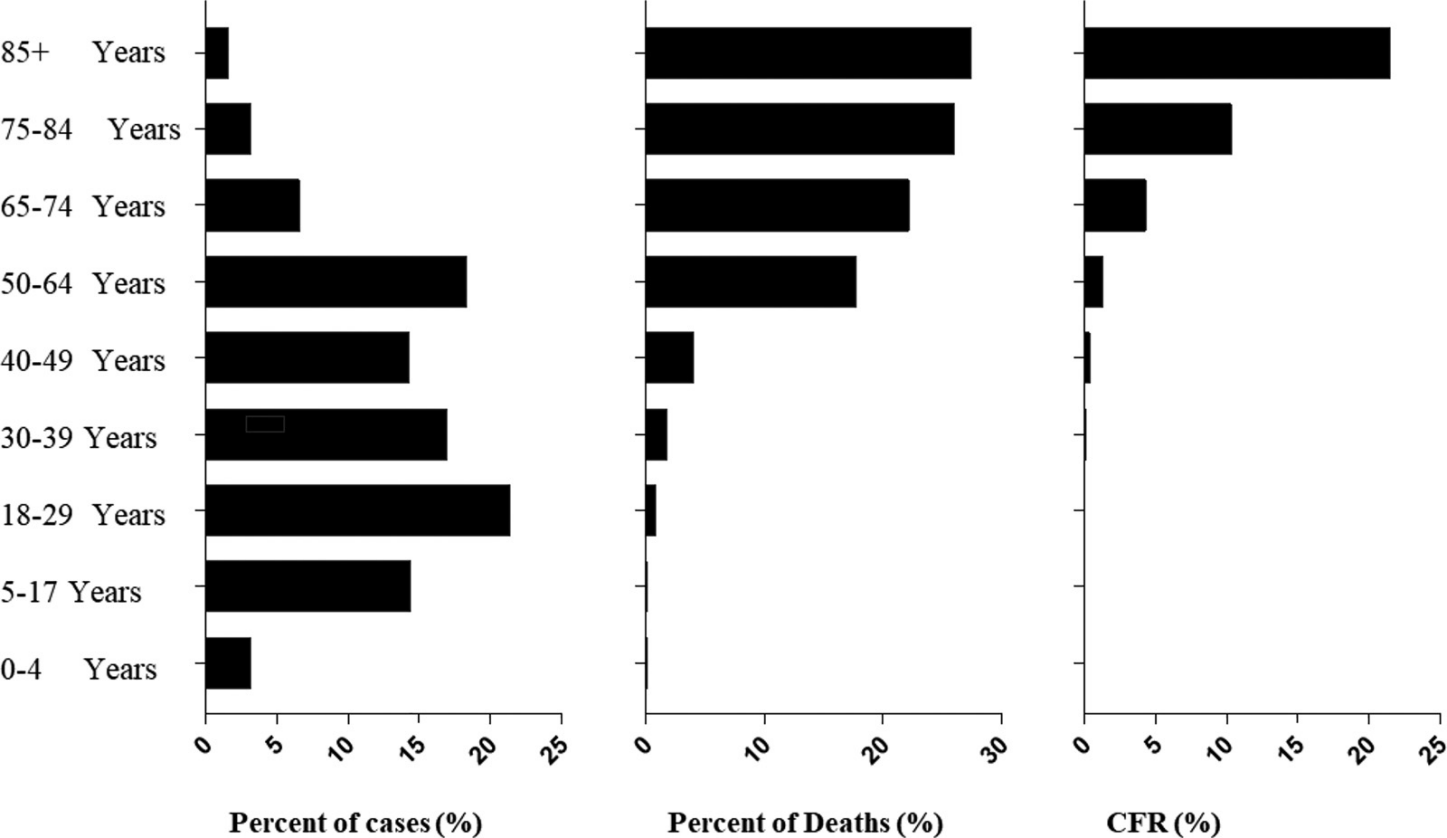
Distribution of COVID-19 cases, deaths, and CFR in different age groups in the United States as of February 18, 2022 (https://covid.cdc.gov/covid-data-tracker/#datatracker-home). (Color version of figure is available online).

Risk factors for CFR of COVID-19 in different countries may include population age, gender, geographical areas, genetic background of the population, and medical and diagnostic conditions. The age of the population may be the most important risk factor for COVID-19 deaths. Based on the COVID Data Tracker in the United States, most COVID-19 cases were reported in younger people (88.5% under 64 years of age; 70.2% under 49 years of age), but the vast majority of deaths (over 93%) occurred in people over 50 years of age or in people with comorbidities or chronic diseases (Fig. 4). The CFR of individuals < 30 years old is less than 0.03%. Importantly, COVID-19 cases seemingly appears to be younger with the sustain of COVID-19 pandemic. In addition, although males and females have the same prevalence, females with COVID-19 seem to be protected from developing a severe disease compared to males. Females contains a more robust antiviral interferon responses, humoral immune responses, and adaptive immunity towards SARS-CoV-2 infections [10].

## 4 Child COVID-19

Initially, COVID-19 was considered an infectious disease for adults, we and others demonstrated that children and neonates are susceptible to COVID-19 but with much less severe symptoms [11], [12], [13]. More and more studies have confirmed that children are susceptible to COVID-19 but exhibit only mild or no symptoms at all. As of February 19, 2022, a total of 78,269,789 cases of COVID-19 cases have been reported in the United States, with children (<12 years old) represented about 10% of all US cases. In states that reported cases of COVID-19, up to 0.01% of all cases of COVID-19 in children were fatal. The question remains to be answered as to whether children should be vaccinated. The US CDC recommends that everyone 12 years of age and older should be vaccinated against COVID-19. The rationale for vaccinating children is that children can become infected with SARS-CoV-2, some may become ill, and infected children may transmit SARS-CoV-2 to others [14]. When deciding whether to vaccinate children younger than 12 years of age, the advantages and disadvantages of the childhood vaccine must be weighed. Multisystem inflammatory syndrome in children (MIS-C) is a rare severe disease that occurs in children who have had COVID-19. The main symptoms of MIS-C include persistent fever and a constellation of symptoms including hypotension, multi-organ dysfunction (eg, cardiac, gastrointestinal, renal, hematologic, dermatologic, and neurologic), and elevated inflammatory response. With only a few reported cases, MIS-C was predominantly reported in Afro-Caribbean descent (75%) and male (62.5%) [15]. The risk factor and pathogenesis of MIS-C are unclear.

## 5 COVID-19 vaccines

To limit the transmission of SARS-CoV-2, various control measures have been recommended, such as mask protection, maintaining social distance, and even cordoning off cities. However, these measures have been implemented in different ways and are unlikely to stop the further spread of SARS-CoV-2 in most countries around the world. Effective preventive vaccines are a top priority in achieving herd immunity, preventing the spread of the disease, and halting the ongoing epidemic. An ideal COVID-19 vaccine is expected to mediate a T_H_1 cell response and promote neutralizing antibody (nAbs) production against SARS-CoV-2 attacks, but reduce negative antibody-dependent enhancement and enhanced respiratory disease [16,17]. The required percentage of immune individuals is estimated to be at least 80% of the population to achieve effective herd immunity [18], which refers to building a threshold of sufficient percentage of a population with adequate immunity, through vaccination or natural infection. As of February 18, 2022, the WHO has reported 339 vaccine candidates in clinical (144) or pre-clinical (195), including inactivated virion vaccines, live attenuated vaccines, protein subunit vaccines, viral vector vaccines, DNA vaccines, and RNA vaccines, more than 7 of which have already been used to vaccinate humans worldwide. The detailed information of COVID-19 vaccines, such as application, side effects, and protection rate, have been described in detail in the literature [17,^19^,20].

COVID-19 vaccine-related efforts and developments are encouraging. COVID-19 vaccines have been included in the immunization programs of many countries. As of February 18, 2022, 54.52% of world population were fully vaccinated and 62.9% of the world population has received at least one dose of a COVID-19 vaccine (Table 1). In addition, considering the COVID-19 cases in the United States, the vaccination program started on December 14, 2020, and by February 18, 2022, about 214.8 million people in the United States (64.7%) and all individuals in Israel were fully vaccinated. Importantly, the vaccination rates in high-income countries are 8 times higher than in the countries of Africa (Table 1). As of February 18, 2022, about 6.56% world population have even been able to fully vaccinate in lower income countries (Table 1), and over 80 countries, especially in Eastern Mediterranean and Africa areas, are struggling to vaccinate their populations to 40% of their population. A comprehensive longitudinal COVID-19 study is needed to ascertain the efficacy, side effects, and duration of protection of COVID-19 vaccines, and to rule out the effect of season on COVID-19 morbidity. To achieve the ambition, additional things need to be noted when vaccines are confirmed as safe and efficacious in phase III or IV clinical trials: People should get equal access and fair distribution of COVID-19 vaccines; governments should promote public acceptance and knowledge of the vaccine; global solidarity is essential and vaccine resources must be pooled and shared to combat pandemics; developed and high-income countries should share vaccines according to fair allocation rules developed by the WHO; people older than 50 years, the high-risk population, need to be vaccinated much more frequently with COVID-19 vaccine when vaccine is insufficient.

**Table 1:** The vaccination rates of different areas worldwide as of February 18, 2022 (https://covid19.who.int/).

Areas	One dose	Fully vaccinated
Global	62.19	54.52
Europe	65.52	60.71
Americas	74.9	64.93
South-East Asia	67.31	53.09
Eastern Mediterranean	43.10	34.54
Western Pacific	83.91	80.91
Africa	12.98	8.67
		
Lower income	10.42	6.56
Lower middle-income	54.17	40.36
Upper middle-income	76.43	70.39
High income	76.66	70.55

## 6 Therapeutic COVID-19 neutralizing antibodies

Many COVID-19 patients are still associated with severe diseases and conditions for which effective therapies are necessary to treat the disease and relieve the clinic burden. Optimal supportive care and dexamethasone are widely used in clinical COVID-19 management. In addition to these non-specific treatments and the preventive function of COVID-19 vaccines, passive nAbs or convalescent plasma treatments may be important in improving the COVID-19 clinical outcomes. Our group has found that COVID-19 patients have recently reported continuously producing nAbs against SARS-CoV-2 with a high titer in a study period of more than one and a half years [21]. In addition, convalescent plasma has been proven to treat infectious diseases for more than a century. Hence, antibodies would be specific to COVID-19 patients or would confer immunity to individuals whose endogenous immune response has not yet been established. To date, like COVID-19 vaccines, many SARS-CoV-2 nAbs have been discovered at various stages of development, showing promising results [22], [23], [24], [25], [26]. In addition, antibody cocktails against SARS-CoV-2, such as REGN-CoV2, may provide more effective antiviral therapy and possibly prevent the emergence of SARS-CoV-2 mutants [27,28].

## 7 SARS-CoV-2 virulence variation, decreasing case fatality of COVID-19, and breakthrough infections

Of particular concern are mutations in the spike protein, especially for the receptor binding domain (RBD) [29], [30], [31]. The first reported SARS-CoV-2 mutation is D614G in the spike protein , which can interact with ACE2 more efficiently and has potentially high infectivity and viral load in the upper respiratory tract of COVID-19 patients, but does not increase disease severity [32,33]. SARS-CoV-2 variants are divided into several categories, including Variants of Concerns (VOCs), Variants of Interests (VOIs), and some other variants under monitoring. VOIs indicate SARS-CoV-2 variants with increasing number of COVID-19 cases and genomic changes that are predicted or known to affect transmissibility, virulence, or the diagnosis of SARS-CoV-2. VOCs indicate that SARS-CoV-2 variants are demonstrated to be associated with high transmissibility, increase in virulence, nAbs resistance, or diagnostic detection failures.

Currently, designated VOCs include Delta (B.1.617 and AY) and Omicron (B.1.1.529 and BA lineages). The Delta variant was identified in India in October 2020. Delta variant is highly contagious, estimated up to 60% more transmissible than the Alpha variant, and exhibits hospital admission risks than the previously reported SARS-CoV-2 variants [34], accompanied with high viral load and transmissibility. Notably, the Delta variant is associated with immune escape from recognition of antibodies that target non-RBD and RBD epitopes of the spike protein [35]. Sera collected from COVID-19 patients or individuals vaccinated by Oxford-ChAdOx1 nCoV-19 and Pfizer BNT162b2 showed reduction in neutralization titers to the Delta variant [36]. Although COVID-19 vaccination could reduce hospital admission and death risks, vaccinated individuals could also mediate transmission of the Delta variant. A single dose of either the AstraZeneca-Oxford ChAdOx1 nCoV-19 or the Pfizer BNT162b2 vaccine showed only 30% effective protection against symptomatic infection with the Delta variant, while a second dose of vaccine could enhance protection rate to 67% and 88%, respectively [37].

Importantly, before the Omicron variant pandemic, the Delta variant was primarily responsible for SARS-CoV-2 outbreaks. An increased number of breakthrough infections have been reported with the emergence of the Delta variant, which has been introduced and discussed in detail [38]. Vaccine breakthrough infections of the Delta variant have been reported in various countries, such as the United States, Israel, Singapore, and India, where different vaccines, including Pfizer BNT162b2, Moderna mRNA-1273, and Covaxin BBV152, were used [39], [40], [41], [42], [43]. For example, breakthrough infections were observed in 2-dose-vaccinated (Pfizer BNT162b2) and booster-vaccinated individuals. Notably, Pfizer BNT162b2 vaccine could protect against Delta variant breakthrough infections, but the protective effects decreased 2 months after vaccination and eventually vanishes 6 months or longer [39].

The first known Omicron variant was discovered in specimens collected in Botswana on November 11, 2021, and in South Africa on November 14, 2021 [44]. The spike protein in the Omicron variant has approximately 30 mutations. Importantly, the Omicron variant quickly spread throughout the world (at least 170 countries as of February 23, 2022), and surpassed the Delta variant as the dominant SARS-CoV-2 variants. First, the Omicron variant is highly contagious and may be 2.8 times more contagious than the Delta variant [45]. In addition, the Omicron variant exhibits a higher reproduction number, which could be 7 or even greater [46], allowing the variant to go unrecognized and easily spread around the world [6]. Second, the Omicron variant prefers to infect the upper respiratory tract and shows lower virulence, resulted in no more severe illness then reported SARS-CoV-2 variants [47,48]. Third, the Omicron variant was able to evade the immune system defenses and resists nAbs in people who had previously been vaccinated or infected with SARS-CoV-2, causing more frequent breakthrough infections than previous variants in recent months [49], [50], [51], [52], [53], [54], [55]. In addition, the variant was resistant to a therapeutic monoclonal antibody [56,57]. Notably, it was predicted that the vaccine-escape capability was about 14 times more efficient than the Delta variant and has an 88% chance to escape current vaccines [45].

Actually, the cause of SARS-CoV-2 breakthrough infections can be divided into 2 parts. On one hand, lower nAb levels may increase the risk of breakthrough infections. On the other hand, the emergence of novel SARS-CoV-2 variants could induce immune escape and promote the risk of breakthrough infections. Breakthrough infections continue to be a major public health concern, raising concerns about vaccine hesitancy and anxiety. The good news is that while the efficacy of current vaccines and nAbs are slowed down, they are still effective in relieving COVID-19 to some content. Notably, breakthrough infection has also been reported in individuals vaccinated with inactivated COVID-19 vaccine [58,59]. As the COVID-19 epidemic progresses, new SARS-CoV-2 variants may emerge worldwide. Further studies are needed to investigate the impact of SARS-CoV-2 variants on infection, transmission, and interactions with natural and/or vaccine-induced immunity. It is also necessary to promote the development of an updated vaccine against the novel SARS-CoV-2 variant.

**Fig. 5:**
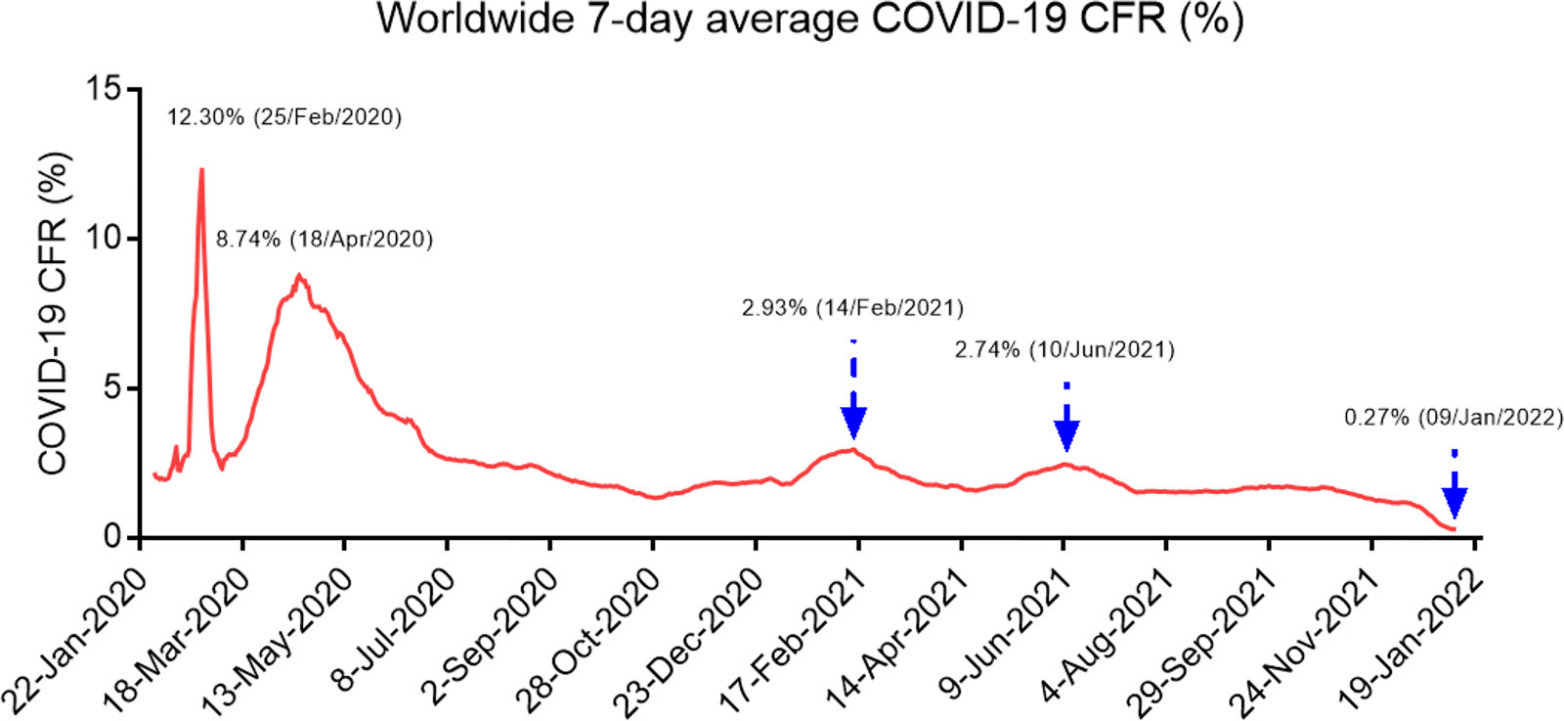
Worldwide 7-day average CFR of COVID-19 from January 22, 2020, to January 9, 2022 (https://www.worldometers.info/coronavirus/). (Color version of figure is available online).

**Fig. 6:**
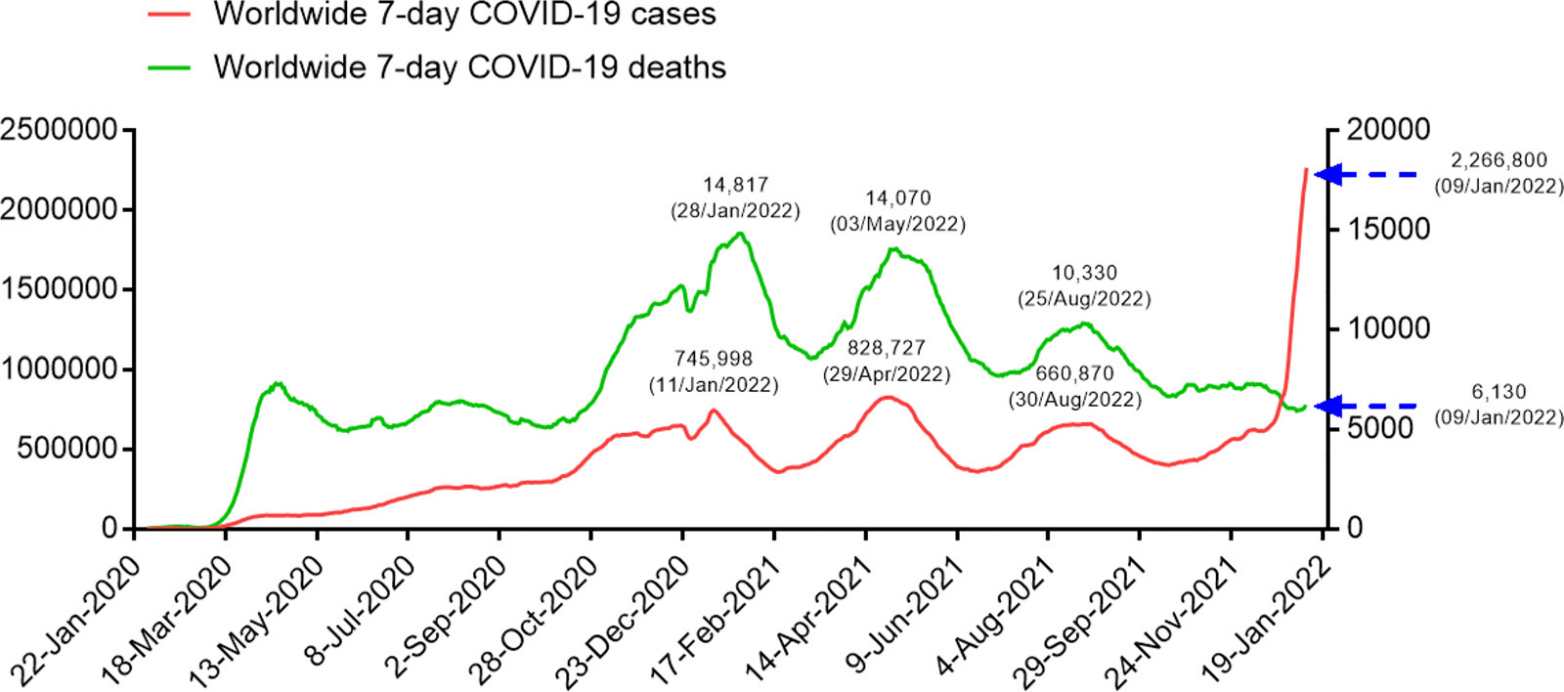
Worldwide 7-day average COVID-19 cases and deaths as of January 9, 2022 (https://www.worldometers.info/coronavirus/). (Color version of figure is available online).

In addition, people are desperate for getting rid of the COVID-19 pandemic, but the goal of eradicating SARS-CoV-2 from much of the world through vaccines is a difficult task, and there is still a long, arduous road ahead. Unlike SARS (CFR 10.88%) or MERS (CFR 34.78%), SARS-CoV-2 exhibits relatively lower pathogenicity but longer latent infection period and higher reproduction number compared to SARS-CoV and MERS-CoV [60]. These characters make SARS-CoV-2 go unrecognized and easy to transmit around the world [6]. As an RNA virus, SARS-CoV-2 tends to accumulate mutations, which is a natural byproduct of viral replication. Although most genomic alterations of the SARS-CoV-2 genome were synonymous, we cannot deny that some mutations may affect virulence. Despite the robust increase of COVID-19 cases since mid-December 2021, the COVID-19 deaths and CFR keeps decreasing (Figs. 5 and 6). The cumulative CFR and 7-day average CFR of COVID-19 has decreased from 6.89% on the April 29, 2020 and 12.3% on the February 25, 2020 to 1.76% and 0.27% January 09, 2022 (Fig. 5). The decline in the CFR of COVID-19 might be related to a number of factors, including better treatment of patients, a decrease in the virulence of the virus variants [16,17], and/or COVID-19 vaccine immunization. Although we do not know whether the mortality rate of SARS-CoV-2 will decrease like other common cold coronaviruses such as HCoV-OC43 and HCoV-HKU1, as the population reaches herd immunity worldwide, the mortality rate of SARS-CoV-2 will continue to decrease [61,62].

## 8 Conclusion

The truth is that we are still trapped in the COVID-19 pandemic phase. Most people around the world are still susceptible to SARS-CoV-2 and could be reinfected by SARS-CoV-2 with the Omicron variant pandemic [63,64]. In the past 2 years, we have exhausted various methods to control COVID-19. Except in China and other East Asian countries, the global control of COVID-19 has very limited effect by merely keeping social distance and wearing masks. Effective and safer COVID-19 vaccines are of great importance in building global herd immunity. Although the impact of the SARS-CoV-2 transmission on the world is complicated, the good news is that the development of COVID-19 vaccines and therapeutic nAbs is well-developed.

Challenges still exists in response to COVID-19 pandemic, include the high mutation rate of the SARS-CoV-2, a lack of funding, personnel, and country commitment, inadequate vaccines in some areas of the world, and uncertainty about the durability of vaccine protection. In the past 2 years, several SARS-CoV-2 variants have emerged that can escape the immune protection of the vaccines based on the original strain of SARS-CoV-2. Therefore, there is an urgent need to develop updated vaccines, similar to the influenza vaccine, to address SARS-CoV-2 mutants. If the developed COVID-19 vaccines provide immunity against emerging SARS-CoV-2 variants, there is optimism that SARS-CoV-2 can be controlled, at least in some regions of the world. SARS-CoV-2 could become endemic in some regions where not enough people are vaccinated. These epidemic areas become difficult factors for the global eradication of SARS-CoV-2. In addition, a vaccine with a short duration of protection will make it difficult to eliminate SARS-CoV-2.

Additionally, many animal populations are also susceptible to SARS-CoV-2, such as ferrets, cats, and dogs [65,66]. Evidence also supports that bats and pangolins are potential SARS-CoV-2 reservoirs [67,68]. However, the SARS-CoV-2 reservoir and the interaction between the SARS-CoV-2 reservoir and humans are still largely unclear. SARS-CoV-2 might come to us again from the potential animal reservoir. Knowledge of zoonotic transmission patterns and increased surveillance of zoonotic diseases are also needed to eradicate SARS-CoV-2 and prevent further spillover events.

Large information about SARS-CoV-2 remains elusive. We do not know whether vaccines could halt the spread of SARS-CoV-2 in a short time or whether SARS-CoV-2 will be eradicated worldwide. It is difficult to predict whether SARS-CoV-2 will be an endemic virus similar to influenza, but it may pose less of a threat over time. It seems impossible to completely control the spread of COVID in the short term. Hopefully, the mortality rate of COVID-19 may continue to decrease and COVID-19 will be eradicated or reduced in some countries through herd immunity if enough people are vaccinated within a year or 2. Stringent preventive measures cannot be neglected before the outbreak of COVID −19 is contained.

## Author contributions

Conceptualization, X-jY. Original draft preparation, C-mZ, X-rQ, l-nY, and X-jY Writing, review and editing, X-jY. Project administration, X-jY. Funding acquisition, X-jY. All authors contributed to the article and approved the submitted version. COVID-related Data was originated from COVID Data Tracker, European center for Disease Prevention and Control, and World Health Organization.

## Declaration of competing interests

The authors declare that they have no known competing financial interests or personal relationships that could have appeared to influence the work reported in this paper.

## Funding sources

This study was supported by the National Natural Science Funds of China (81971939 and 31570167) and the Fundamental Research Funds for the Central Universities (2042021kf0046). The funders had no role in the study design, data collection and analysis, decision to publish, or the preparation of the manuscript.
